# Virologic outcomes on Dolutegravir-based therapy among children and adolescents living with HIV in Thailand: findings from a national registry database

**DOI:** 10.1016/j.lansea.2026.100782

**Published:** 2026-05-20

**Authors:** Sirinya Teeraananchai, Wipaporn Natalie Songtaweesin, Suvaporn Anugulruengkitt, Suthat Chottanapund, Intira Jeannie Collins

**Affiliations:** a Department of Statistics, Faculty of Science, Kasetsart University, Bangkok, Thailand; b Special Research Incubator Unit for Innovations in Statistics and Digital Health, Kasetsart University, Bangkok, Thailand; c School of Global Health, Faculty of Medicine, Chulalongkorn University, Bangkok, Thailand; d Center of Excellence for Pediatric Infectious Diseases and Vaccines, Faculty of Medicine, Chulalongkorn University, Bangkok, Thailand; e Division of Infectious Diseases, Department of Pediatrics, Faculty of Medicine, Chulalongkorn University, Bangkok, Thailand; f Department of Disease Control, Ministry of Public Health, Nonthaburi, Thailand; g UCL Innovative Clinical Trials Unit, Institute of Clinical Trials & Methodology, University College London, London, UK

**Keywords:** HIV, Children, Adolescents, DTG, Viral suppression, Virologic failure

## Abstract

**Background:**

Dolutegravir (DTG)-based antiretroviral therapy (ART) is recommended for initial and subsequent ART for children and adolescents living with HIV (CALHIV). We evaluated virologic outcomes after starting DTG-based ART using the National AIDS Program (NAP) database in Thailand.

**Methods:**

We conducted a cohort study including CALHIV aged <18 years at DTG start, with at least one viral load (VL) measurement after starting DTG-based ART. Outcomes were: (i) viral suppression (VS) (defined as VL < 1000 and <50 copies/mL), (ii) episodes of viremia (≥1 VL ≥ 1000 copies/mL), and (iii) among those with ≥2 VL measurements, confirmed virologic failure (VF) (two consecutive VL ≥ 1000 copies/mL). Cox regression models were used to assess factors associated with viremia and VF, and generalized estimating equations models to assess factors associated with rates of viremia over time.

**Findings:**

Of 2395 CALHIV included, 49% were female and 69% were ART-experienced at DTG start. Median age at DTG start was 15 years (Interquartile range [IQR] 12–17). Over a median 1.55 years [1.05–1.92] on DTG and a median of 2 [1–2] VL measurements per participant, 84.3% had VS < 1000 copies/mL and 75.9% <50 copies/mL throughout follow-up; 15.7% experienced viremia. Among those with ≥2 VL measurements available, 7.1% had confirmed VF. In adjusted models, female sex and being ART-experienced with detectable VL at DTG start were associated with a significantly higher risk of viremia and VF. Rates of episodic viremia increased over time and varied by age, with higher rates among those aged < 6 years and 12–15 years at DTG start compared to children aged 6–<12 year.

**Interpretation:**

Most CALHIV achieved viral suppression on DTG. Overall risk of virologic failure was low but was substantially higher among ART-experienced individuals with detectable VL at DTG start, underscoring the importance of close monitoring and targeted adherence support for vulnerable subgroups.

**Funding:**

Kasetsart University Research and Development Institute, Kasetsart University, Thailand.

**Research in context**

**Evidence before this study**

We searched PubMed and Google Scholar for studies published up to May 2025 using the terms “dolutegravir,” “children,” “adolescents,” “HIV,” and “virologic outcomes.” While there are emerging evidence on virologic outcomes on dolutegravir (DTG) in children and adolescents living with HIV (CALHIV) in routine-care settings, including large cohorts in sub-Saharan Africa and Europe, there remains a critical gap in national level data and in-depth analysis of virologic failure (VF) and associated factors, especially within a Southeast Asian setting.

**Added value of this study**

This is the first national-level study to evaluate real-world virological outcomes of DTG-based antiretroviral therapy (ART) among CALHIV. Using Thailand’s National AIDS Program database, we assessed outcomes in 2395 CALHIV receiving DTG-based ART. Over a median of 1.6 years (IQR 1.05–1.92) of DTG-based ART and median of 2 (IQR 1–2) viral load (VL) measurements per participant, 84.3% had viral suppression (VS) at VL < 1000 copies/mL and 75.9% at VL < 50 copies/mL throughout their follow-up time on DTG. This study demonstrated high rates of VS among CALHIV, while highlighting a significantly higher risk of virologic failure among those ART-experienced with detectable VL at DTG start. Large-scale cohort studies from sub-Saharan Africa have similarly reported high rates of viral suppression among CALHIV on DTG, but these were largely conducted in research-enriched or programme-specific settings. By contrast our analysis draws on a comprehensive national database in a middle-income Asian country, reflecting a distinct epidemiological profile, health system, and ART delivery model. This approach demonstrates the generalisability of DTG effectiveness beyond the African context and provides robust, policy-relevant evidence to inform national treatment guidelines, support the global scale-up of DTG for CALHIV, and identify subgroups at higher risk of virologic failure who may benefit from enhanced monitoring and adherence support.

**Implications of all the available evidence**

DTG-based ART is effective for most CALHIV, supporting its continued scale-up in national programs. However, findings highlight the need for targeted monitoring and adherence support among sub-groups at higher risk of virologic failure to ensure the durability of this regimen.

## Introduction

Globally, more than 1.5 million children and adolescents are living with HIV (CALHIV), the majority of whom reside in low- and middle-income countries.^1^^,^^2^ Antiretroviral therapy (ART) has substantially reduced HIV-related mortality and improved life expectancy among people living with HIV.^3^, ^4^, ^5^ Despite advances in ART, viral suppression among CALHIV remains suboptimal compared to adults.^6^^,^^7^ Dolutegravir (DTG)-based ART is the preferred regimen across all age groups because of its potency, high genetic barrier to resistance, once-daily dosing, and favourable safety profile.^8^ Emerging data from routine-care settings in a large paediatric cohort (>9000 participants) in eastern and southern Africa report high levels of viral suppression (93% suppressed <1000 copies/mL at last visit) and low mortality (0.8%) during the study period.^9^ Similarly, a small cohort study in Thailand (n < 100) reported high levels of viral suppression (90% suppressed <200 copies/mL) at one year after starting DTG among youth living with HIV.^10^

In the pre-DTG era, approximately one in five CALHIV receiving non-nucleoside reverse transcriptase inhibitor (NNRTI)– or protease inhibitor (PI)–based ART required a regimen switch within three years due to treatment failure. The scale-up of DTG-based ART has included its use as first-line treatment for ART-naïve individuals, as well as second or subsequent-line therapy for ART-experienced individuals with virologic failure, and as treatment substitution for those with viral suppression on ART.^11^ However, evidence on virologic failure (VF) and its associated factors among individuals receiving DTG in routine care remains limited, particularly national-level data among CALHIV in Asia.^10^^,^^12^, ^13^, ^14^, ^15^

Thailand provides free HIV testing and treatment through the Universal Health Coverage program (UHC).^16^, ^17^, ^18^ Approximately 98% of CALHIV aged <18 years receive care within this programme, with a small minority attending private facilities.^11^^,^^16^ DTG was incorporated into the Thai national HIV treatment guidelines in 2021 as the preferred first- and subsequent-line regimen for children and adults aged ≥3 years weighing ≥15 kg, and was subsequently extended to children aged <3 years in 2023 using dispersible tablet formulations. Selected centres had earlier access to DTG from 2017 onwards through early implementation and special programme access.^19^ This study uses routine care data from the National AIDS Program (NAP) databases to evaluate virologic outcomes among CALHIV receiving DTG-based regimens across Thailand, with the aim of informing future clinical care in this population.

## Methods

This observational cohort study included all CALHIV receiving HIV care under Thailand’s UHC program and recorded in the NAP database up to August 2024. Inclusion criteria for this analysis were: (i) age < 18 years at start of DTG-based ART (hereafter ‘DTG start’); and (ii) at least one VL measurement after DTG start. CALHIV who initiated ART prior to entry in the NAP database (e.g., those initiated ART abroad or through private healthcare providers) were excluded due to incomplete ART history and clinical data.

De-identified individual-level data were extracted from the NAP database,^16^ including demographic, clinical, ART and laboratory data from first entry into NAP and all subsequent visits. The database is linked to the National Death Registry, allowing near real-time updating of vital status. ART is prescribed in accordance with the Thai National HIV Treatment Guidelines.^16^^,^^20^^,^^21^ As part of routine HIV care, CD4 counts are measured every six months and VL testing is conducted six months after ART initiation and then annually.^19^^,^^20^ The analytic dataset was reviewed for internal consistency, duplication and missingness data and logical checks were performed to ensure chronological consistency.

Characteristics at ART initiation and at DTG start were described overall and stratified by ART history and VL status at DTG start, based on the closest VL within 12 months prior to DTG start: (i) ART naïve; (ii) ART-experienced and suppressed (VL < 50 copies/mL); (iii) ART-experienced with detectable VL (VL ≥ 50 copies/mL); and (iv) ART-experienced with unknown VL.

Mode of HIV acquisition is not routinely captured and was inferred using age at ART initiation as a proxy: individuals initiating ART aged < 15 years were assumed to have perinatally acquired HIV, and those aged ≥ 15 years behaviourally acquired HIV. Opportunistic infection (OI) status was recorded as a binary variable (yes/no) reflecting any documented OI diagnosis prior to or at ART initiation or DTG-start. HIV disease stage was classified according to WHO clinical staging as asymptomatic HIV (stages 1–2), symptomatic HIV(stage 3), or AIDS (stages 4).

Baseline CD4 count at DTG start was defined as the value closest within 12 months prior to and up to 1 month after DTG start. Follow-up was censored at the earliest of death, last clinic visit or DTG discontinuation. Mortality was ascertained from the National Death Registry. Loss to follow-up (LTFU) was defined as no clinic visit for more than 12 months after DTG start.

Virological outcomes were:

(i) Viral suppression (VS), defined as VL < 50 copies/mL (as per Thai guidelines)^19^ and VL < 1000 copies/mL (WHO guidelines)^22^ during follow-up on DTG;
(ii) Viremia, defined as ≥1 VL measurement ≥1000 copies/mL occurring ≥3 months after DTG start, consistent with Thai guidelines thresholds for adherence assessment and repeat testing;
(iii) confirmed virologic failure (VF), defined ≥2 consecutive VL measurements ≥1000 copies/mL, as per standard research definitions and WHO recommendations^2^; and
(iv) Recurrent viremia, defined as repeated episodes of VL ≥ 1000 copies/mL over longitudinal follow-up.

Factors associated with outcomes (ii)-(iv) were explored.

### Statistical analysis

Characteristics at ART initiation and DTG start were summarised using descriptive statistics. Categorical characteristic were compared across ART/VL status groups using Pearson’s Chi-square test, and continuous characteristics using the Kruskal–Wallis test. VF rates were calculated per 100 person-years (PYs) of follow-up. Kaplan–Meier graphs were used to estimate the cumulative probability of viremia and confirmed VF, stratified by sex, ART/VL status and CD4 at DTG start.

Cox proportional hazard model was used assess factors associated with time to first viremia and confirmed VF. Recurrent viremia was analysed using generalized estimating equations (GEE) with a Poisson distribution, log link function, and exchangeable correlation matrix to estimate population-averaged rates over time, accounting for within-individual correlation of repeated VL measurements. This approach incorporates all available visits and reduces bias due to censoring or dropout.

Covariates assessed included the following characteristics at DTG start: age, sex at birth, ART/VL status, DTG regimen, CD4 count, geographic region, calendar year and history of OIs. CD4 count at DTG start was included as a *baseline covariate* in Cox models, categorised into predefined strata. For GEE analyses, CD4 count was treated as a *time-varying covariate*, based on the most recent measurement available at each follow-up visit. Complete-case analysis was used for regression models, with missing CD4 values at baseline or follow-up categorised as “unknown”. No imputation of missing data was performed. Variables with a p-value < 0.10 in univariable analysis were considered for multivariable models. Analyses were performed using SAS version 9.4 (SAS Institute Inc., Cary, NC) and Stata version 18 (StataCorp, College Station, TX).

### Ethics statement

The study was approved by the Kasetsart University Research Ethics Committee (KUREC-HSR67/043), Thailand. The requirement for informed consent was waived because this study involved secondary analysis of routinely collected programmatic data. All data were de-identified by the National Health Security Office prior to analysis.

### Role of the funding source

This study was supported by the Kasetsart University Research and Development Institute (KURDI), Kasetsart University, Thailand (Grant No. FF(KU-SRIU)14.68; 2025–2026). The funder had no role in study design, data collection, data analysis, data interpretation, or writing of the report.

## Results

### Study population

Of 470,471 people living with HIV who initiated ART between January 2008 and August 2024 in the NAP database, 12,363 were CALHIV. Of these, 9199 (74%) either remained on non-DTG regimens or transitioned to DTG at age≥18 years and were excluded. Of the 3164 (26%) CALHIV who received DTG aged <18 years, 682 (22%) had no VL measurements after DTG start (including 522 with <6 months of follow-up), and 87 (3%) had incomplete ART history; therefore, 2395 (76%) were included in the analysis (Supplementary Fig. S1).

Characteristics at ART initiation and DTG start are shown in Table 1. Overall, 51% were male. A total of 1605 (67%) initiated ART aged <15 years and were categorised as having perinatally acquired HIV, Median (interquartile range [IQR]) age at ART initiation was 8 years,^2^, ^3^, ^4^, ^5^, ^6^, ^7^, ^8^, ^9^, ^10^, ^11^, ^12^, ^13^, ^14^, ^15^, ^16^ Median CD4 count at DTG start was 529 cells/mm^3^ [ 318–778].

**Table 1:** Characteristics by ART initiation and at DTG start, overall and by ART and viral load status at DTG start.

Characteristics	ART naive	ART experienced with VL < 50 copies/mL	ART experienced with VL ≥ 50 copies/mL	ART experienced with unknown VL	Total
	736 (31)	1118 (47)	382 (16)	159 (6)	2395 (100)
At ART initiation					
Sex at birth					
Male	442 (60)	556 (50)	157 (41)	74 (47)	1229 (51)
Female	294 (40)	562 (50)	225 (59)	85 (53)	1166 (49)
Age, years	16 [15–17]	4 [1–9]	5 [1–10]	3 [0–9]	8 [2–16]
<6	21 (3)	632 (57)	211 (55)	99 (62)	963 (40)
6–<12	39 (5)	274 (25)	101 (26)	28 (18)	442 (18)
12–<15	70 (10)	80 (7)	34 (9)	16 (10)	200 (8)
≥15	606 (82)	132 (12)	36 (9)	16 (10)	790 (33)
Mode of HIV acquisition					
Perinatal	130 (18)	986 (88)	346 (91)	143 (90)	1605 (67)
Non-Perinatal	606 (82)	132 (12)	36 (9)	16 (10)	790 (33)
Calendar year					
2008–2018	16 (2)	828 (74)	268 (70)	128 (81)	1240 (52)
2019–2021	29 (4)	237 (21)	87 (23)	29 (18)	382 (16)
2022–2024	691 (94)	53 (5)	27 (7)	2 (1)	773 (32)
CD4, cells/mm^3^	383 [232–561]	519 [220–1065]	408 [135–812]	602 [280–1269]	440 [213–802]
<200	134 (18)	246 (22)	117 (31)	23 (14)	520 (22)
200–<350	176 (24)	131 (12)	38 (10)	16 (10)	361 (15)
350–<500	164 (22)	125 (11)	44 (12)	22 (14)	355 (15)
≥500	224 (30)	531 (47)	148 (39)	80 (50)	983 (41)
Unknown	38 (5)	85 (8)	35 (9)	18 (11)	176 (7)
First regimen					
NNRTI + NRTI	0 (0)	882 (79)	290 (76)	114 (72)	1286 (54)
PIs + NRTI	0 (0)	227 (20)	90 (24)	42 (26)	359 (15)
DTG-based	736 (100)	0 (0)	0 (0)	0 (0)	736 (31)
Others	0 (0)	9 (1)	2 (1)	3 (2)	14 (1)
HIV stages					
Asymptomatic HIV	501 (68)	672 (60)	207 (54)	105 (66)	1485 (62)
Symptomatic HIV	65 (9)	86 (8)	23 (6)	8 (5)	182 (8)
AIDS	170 (23)	360 (32)	152 (40)	46 (29)	728 (30)
At DTG start					
Age, years	16 [15–17]	14 [11–16]	14 [11–16]	14 [11–16]	15 [12–17]
<6	18 (2)	52 (5)	38 (10)	11 (7)	119 (5)
6–<12	39 (5)	277 (25)	69 (18)	36 (23)	421 (18)
12–<15	73 (10)	320 (29)	94 (25)	49 (31)	536 (22)
≥15	606 (82)	469 (42)	181 (47)	63 (40)	1319 (55)
CD4, cells/mm^3^	368 [225–540]	724 [530–968]	422 [173–687]	714 [560–923]	540 [324–805]
<200	134 (18)	22 (2)	87 (23)	0 (0)	243 (10)
200–<350	171 (23)	36 (3)	43 (11)	3 (2)	253 (11)
350–<500	154 (21)	101 (9)	38 (10)	11 (7)	304 (13)
≥500	199 (27)	601 (54)	130 (34)	61 (38)	991 (41)
Unknown	78 (11)	358 (32)	84 (22)	84 (53)	604 (25)
Opportunistic infection(s)					
Yes	23 (3)	105 (9)	39 (10)	22 (14)	189 (8)
No	713 (97)	1013 (91)	343 (90)	137 (86)	2206 (92)
Calendar year					
2017–2018	11 (1)	5 (0)	4 (1)	6 (4)	26 (1)
2019–2021	28 (4)	52 (5)	32 (8)	17 (11)	129 (5)
2022–2024	697 (95)	1061 (95)	346 (91)	136 (86)	2240 (94)
First DTG regimen					
DTG + NRTIs	736 (100)	1104 (99)	357 (93)	153 (96)	2350 (98)
DTG + PIs( ± NRTIs)	0 (0)	14 (2)	25 (7)	6 (4)	45 (2)
Viral load, log 10 VL	1.41 [1.30–1.65]	1.30 [1.30–1.60]	3.70 [2.48–4.87]	NA	1.43 [1.30–1.69]
Duration on ART, years	–	7.38 [3.62–10.78]	6.89 [2.82–10.42]	7.99 [3.68–11.40]	4.06 [0.00–9.38]

Characteristics are summarized as n (%) or median [IQR]. Abbreviations: ART, antiretroviral therapy; DTG, Dolutegravir; VL, viral load; NRTIs, nucleoside reverse transcriptase inhibitors; NNRTIs, non-nucleoside reverse transcriptase inhibitors; PI, protease inhibitor; Presented as n (%) for categorical data and median [interquartile range, IQR] for continuous data. Opportunistic infections (OIs) reflect diagnoses recorded prior to or at the time of DTG start.

Median follow-up duration after DTG start was 1.55 years [ 1.05–1.92]. The median number of VL measurements was 2 [1–2 or 1–3] across ART-naïve and ART-experienced subgroups. During follow-up, 73 (3%) participants were LTFU and 23 (1%) died. The overall mortality rate was 0.18 per 100 person-years (PYs) (95% CI: 0.12–0.27).

#### Virological outcomes

**Fig. 1:**
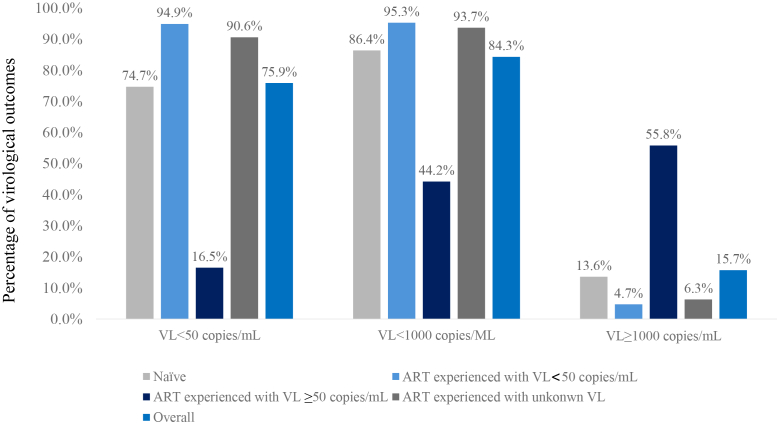
**Percentage with viral suppression or viremia over their observed time on DTG, overall and by ART and viral load status at DTG start.** Note: VL; viral load, ART; antiretroviral therapy.

**Fig. 2:**
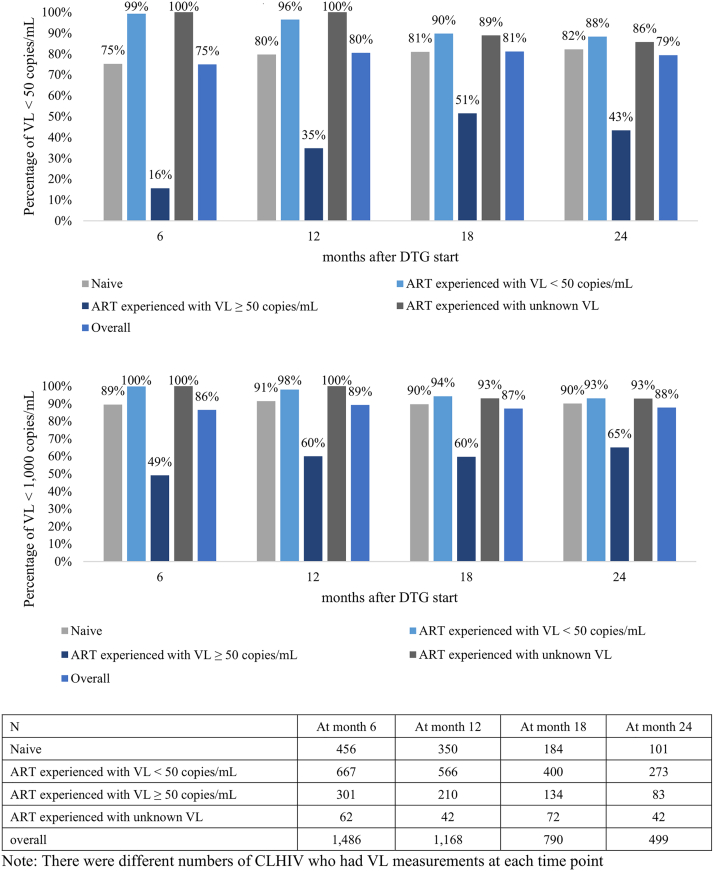
**Percentage with viral suppression (VL < 50 copies/mL, VL < 1000 copies/mL) after DTG start at month 6, 12, 18 and 24 by ART and VL status at DTG start.** Note: There were different numbers of CLHIV who had VL measurements at each time point.

Overall, 75.9% of CALHIV had VS < 50 copies/mL and 84.3% were suppressed <1000 copies/mL during follow-up on DTG, while 15.7% had at least one VL ≥ 1000 copies/mL (Fig. 1) (Fig. 2).

#### Viremia and associated factors

**Fig. 3:**
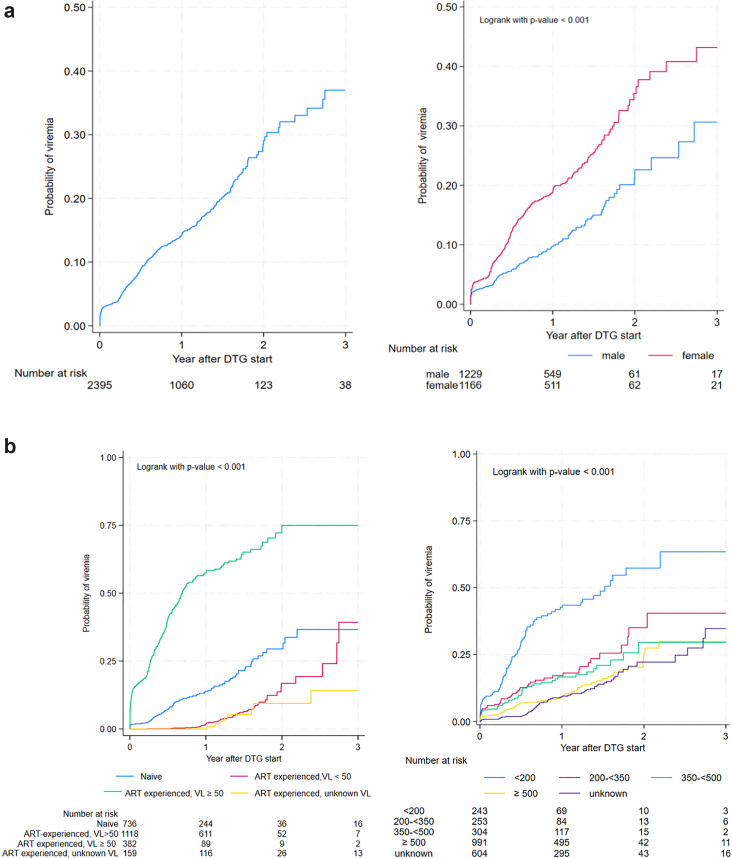
**Cumulative incidence of viremia; a) overall and by sex at birth and b) by ART and VL status and CD4 at DTG start**.

Overall, 375 of 2395 (15.7%) CALHIV experienced viremia (VL ≥ 1000 copies/mL) on DTG. Median time to first viremic episode was 6 [ 3–12] months. The crude rate of viremia was 15.86 per 100 PYs (95% CI: 14.33–17.55) over 2364 PYs of follow-up. The cumulative incidence of viremia was 14% (95% CI: 13–16), 29% (95% CI: 25–33) and 37% (95% CI: 31, 44) by 1, 2 and 3 years, respectively (Fig. 3a).

By 1 year, viremia incidence was highest among ART-experienced individuals with detectable VL at DTG start (57%, 95% CI: 52–63), those with CD4<200 cells/mm^3^ at DTG start (55%, 95% CI: 44–69), and females (20%, 95% CI: 17–24) (log rank test all p < 0.0001) (Fig. 3b).

Table 2 presents rates of viremia and results from univariable and multivariable models. In the multivariable analysis, ART/VL status at DTG start was strongly associated with increased hazard of viremia. Compared with ART-naïve participants, those ART-experienced with detectable VL at DTG start had a five-fold increase in hazard [adjusted hazard ratio (aHR) 5.20, 95% CI: 4.02–6.72], while those ART-experienced and suppressed at DTG start had a significantly lower hazard of viremia (aHR 0.32, 0.23–0.46). Females sex was associated with a 71% increased risk of viremia compared to males (aHR 1.71, 95% CI: 1.38–2.13). Adolescents aged 12–15 years at DTG start had nearly double the hazard compared to children aged 6–<12 years (aHR 1.97, 95% CI: 1.36–2.86). Severe immunosuppression at DTG start (CD4 counts <200) ells/mm^3^) had a two-fold increase in hazard of viremia compared to the reference group of CD4 counts ≥500 cells/mm^3^ (aHR 2.13, 95% CI: 1.59–2.84). More recent calendar year (2022–24) of DTG start was associated at higher risk of viremia compared to 2017–2018 (aHR 4.58, 1.68–12.53). DTG regimen was not associated with viremia.

**Table 2:** Rates of viremia and confirmed virologic failure (VF) and associated factors.

Characteristics at DTG start	Viremia rate per 100 PYs (95% CI)	Viremia (N = 2395)				Confirmed VF rate per 100 PYs (95% CI)	Confirmed VF (N = 1290)			
		Univariable		Multivariable			Univariable		Multivariable	
		HR (95% CI)	p-value	aHR (95% CI)	p-value		HR (95% CI)	p-value	aHR (95% CI)	p-value
Sex at birth			<0.001		<0.001			<0.001		0.01
Male	11.0 (9.3–13.0)	1 (reference)		1 (reference)		2.6 (1.8–3.9)	1 (reference)		1 (reference)	
Female	21.0 (18.5–23.9)	1.91 (1.55–2.36)		1.71 (1.38–2.13)		6.7 (5.3–8.6)	2.56 (1.62–4.06)		1.90 (1.18–3.05)	
Age, years			0.03		0.002			0.18		0.18
<6	24.0 (16.5–34.7)	2.58 (1.59–4.18)		2.01 (1.22–3.29)		18.3 (11.2–29.9)	1.38 (0.44–4.35)		1.10 (0.34–3.56)	
6–<12	9.3 (6.8–12.6)	1 (reference)		1 (reference)		9.4 (6.7–13.3)	1 (reference)		1 (reference)	
12–<15	17.8 (14.6–21.7)	1.92 (1.33–2.76)		1.97 (1.36–2.86)		17.8 (14.2–22.3)	2.08 (1.04–4.17)		1.92 (0.94–3.92)	
≥15	16.5 (14.4–18.9)	1.79 (1.28–2.5)	0.06	1.60 (1.13–2.26)		16.5 (14.1–19.4)	1.67 (0.86–3.22)		1.18 (0.60–2.33)	
ART and VL status			<0.001		<0.001			<0.001		<0.001
ART Naïve	15.2 (12.5–18.6)	1 (reference)		1 (reference)		2.5 (1.4–4.5)	1 (reference)		1 (reference)	
ART experienced with VL < 50 copies/mL	4.2 (3.2–5.6)	0.27 (0.20–0.38)		0.32 (0.23–0.46)		1.9 (1.1–3.1)	0.26 (0.10–0.67)		0.31 (0.12–0.83)	
ART experienced with VL ≥ 50 copies/mL	82.4 (72.0–94.3)	5.18 (4.08–6.58)		5.20 (4.02–6.72)		23.2 (18.3–29.3)	9.20 (4.97–17.0)		7.94 (4.21–14.98)	
ART experienced with unknown VL	4.2 (2.2–7.9)	0.28 (0.15–0.54)		0.37 (0.19–0.73)		1.2 (0.3–4.9)	0.43 (0.10–1.94)		0.55 (0.12–2.57)	
CD4 count (cells/mm^3^)			<0.001		<0.001			<0.001		<0.001
<200	52.3 (42.9–63.9)	4.46 (3.39–5.85)		2.13 (1.59–2.84)		18.9 (13.3–26.7)	10.24 (5.61–18.67)		3.86 (2.04–7.29)	
200–<350	19.6 (14.6–26.3)	1.76 (1.25–2.49)		1.15 (0.80–1.66)		4.1 (2.0–8.2)	2.31 (0.99–5.40)		1.30 (0.53–3.14)	
350–<500	17.4 (13.1–23.2)	1.49 (1.06–2.09)		1.26 (0.88–1.79)		7.3 (4.4–11.9)	3.75 (1.88–7.50)		2.53 (1.23–5.20)	
≥500	11.5 (9.6–13.8)	1 (reference)		1 (reference)		1.1 (3.1–3.1)	1 (reference)		1 (reference)	
Unknown	10.4 (8.2–13.0)	0.9 (0.67–1.21)		0.76 (0.56–1.02)		3.8 (2.4–5.9)	1.88 (0.96–3.66)		1.35 (0.69–2.66)	
Having OIs			0.59					0.59		
Yes	17.6 (12.5–24.7)	1.11 (0.77–1.58)				3.9 (1.7–8.8)	0.80 (0.35–1.84)			
No	15.7 (14.1–17.4)	1 (reference)				4.8 (3.9–5.9)	1 (reference)			
First DTG regimen			0.01		0.73			0.42		
DTG + NRTIs	15.5 (13.9–17.2)	1 (reference)		1 (reference)		4.6 (3.7–5.7)	1 (reference)			
DTG + PIs ( ± NRTIs)	50.1 (29.0–86.2)	1.98 (1.22–3.20)		0.92 (0.56–1.51)		13.7 (5.1–36.5)	1.49 (0.60–3.74)			
Year			0.021		<0.001			0.25		
2017–2018	4.7 (2.1–10.6)	1 (reference)		1 (reference)		1.5 (0.3–6.3)	1 (reference)			
2019–2021	14.5 (10.4–20.2)	2.53 (0.96–6.66)		3.33 (1.21–9.18)		5.7 (3.3–9.6)	3.09 (0.62–15.29)			
2022–2024	16.7 (15.0–18.6)	3.16 (1.21–8.26)		4.58 (1.68–12.53)		4.8 (3.8–6.1)	3.26 (0.65–16.43)			

HR, hazard ratio; aHR, adjusted hazard ratio, 95% CI, 95% confidence interval, ART, antiretroviral therapy; PI, protease inhibitor; DTG, Dolutegravir; NRTIs, nucleoside reverse transcriptase inhibitors; VL, viral load; OI, opportunistic infection.

#### Virologic failure and associated factors

Among 1290 CALHIV who had ≥2 VL measurements, the characteristics were broadly comparable to those included in the main analysis (Supplementary Table S2 Cumulative incidence of VF by 1, 2 and 3 years was 4% (95% CI: 3, 5), 11% (95% CI: 9, 14) and 14% (95% CI: 10, 20), respectively (Supplementary Table S3, Supplementary Fig. S3). In the multivariable analysis, ART/VL status at DTG start was the strongest predictor of VF (Table 2).

#### Recurrent viremia over time and associated factors

Table 3 shows the multivariable model of factors associated with recurrent viremia. ART-experienced individuals with detectable VL at DTG start had nearly three-fold higher rate of recurrent viremia [adjusted incidence rate ratio (aIRR) 3.13, 95% CI: 2.42–4.05], while those ART-experienced and suppressed at DTG start had substantially lower rates (aIRR 0.31, 95% CI: 0.22–0.45) compared with ART-naïve participants. Females had a 48% higher rate than males (aIRR 1.48, 95% CI: 1.20–1.83). Compared with children aged 6–<12 years, higher rates were observed among those aged <6 years (aIRR 1.67, 95% CI: 1.03–2.71) and adolescents aged 12–<15 years (aIRR 1.47, 95% CI: 1.03–2.11).

**Table 3:** Factors associated with rates of recurrent viremia over time.

Characteristics at DTG start (N = 2395)	VL ≥ 1000 copies/mL			
	Univariable		Multivariable	
	IRR (95% CI)	p-value	aIRR (95% CI)	p-value
Sex at birth		<0.001		<0.001
Male	1 (reference)		1 (reference)	
Female	1.95 (1.55–2.44)		1.48 (1.20–1.83)	
Age, years		0.002		0.04
<6	2.51 (1.49–4.24)		1.67 (1.03–2.71)	
6–<12	1 (reference)		1 (reference)	
12–<15	1.94 (1.31–2.89)		1.47 (1.03–2.11)	
≥15	1.74 (1.21–2.51)		1.11 (0.79–1.56)	
ART and VL status at DTG initiation		<0.001		<0.001
ART Naïve	1 (reference)		1 (reference)	
ART experienced with VL < 50 copies/mL	0.28 (0.20–0.41)		0.31 (0.22–0.45)	
ART experienced with VL ≥ 50 copies/mL	4.39 (3.47–5.55)		3.13 (2.42–4.05)	
ART experienced with unknown VL	0.41 (0.20–0.81)		0.43 (0.21–0.87)	
Year		0.02		0.06
2017–2018	0.58 (0.18–1.91)		0.51 (0.19–1.36)	
2019–2021	1.63 (1.14–2.32)		1.06 (0.76–1.46)	
2022–2024	1 (reference)		1 (reference)	
Current CD4 count (cells/mm3)		<0.001		<0.001
<200	9.86 (7.52–12.92)		3.72 (2.78–4.97)	
200–<350	4.59 (3.35–6.30)		2.92 (2.11–4.05)	
350–<500	2.22 (1.59–3.10)		1.74 (1.24–2.44)	
≥500	1 (reference)		1 (reference)	
Unknown	1.75 (1.33–2.31)		1.65 (1.25–2.17)	
Having OIs		0.65		
Yes	1.09 (0.74–1.61)			
No	1 (reference)			
First DTG regimen		0.002		0.15
DTG + NRTIs	1 (reference)		1 (reference)	
DTG + PIs ( ± NRTIs)	2.25 (1.34–3.79)		0.99 (0.61–1.61)	

IRR, Incidence rate ratio; aIRR, adjusted Incidence rate ratio, 95% CI, 95% confidence interval, ART, antiretroviral therapyPI, protease inhibitor; DTG, Dolutegravir; NRTIs, nucleoside reverse transcriptase inhibitors; VL, viral load; OI, opportunistic infection.

CD4 was included as a time-updated covariate in the models.

Time-updated CD4 count showed a strong dose–response relationship. Compared with CD4 ≥500 cells/mm^3^, CD4 <200 cells/mm^3^ was associated with more than a three-fold increase in viremia rates (aIRR 3.72, 95% CI: 2.78–4.97). Moderate immunosuppression with CD4 200–<350 (aIRR 2.92, 95% CI: 2.11–4.05), and CD4 350–<500 (aIRR 1.74, 95% CI: 1.24–2.44) were also associated with higher rates. Calendar year of DTG start, and DTG regimen were not associated with recurrent viremia.

## Discussion

This nationwide cohort study provides real-world evidence on virologic outcomes of DTG-based ART among CALHIV in Thailand. Overall, 84% achieved viral suppression during follow-up, which is below the global target of ≥95%, although this level was achieved among the subgroup ART-experienced and suppressed at DTG start. Outcomes were substantially poorer among ART-experienced individuals with detectable VL at DTG start, over half of whom experienced VL ≥ 1000 copies/mL during follow-up and had a markedly higher risk of both viremia and confirmed VF. In multivariate analysis, female sex and severe immunosuppression at DTG start were independently associated with increased risk of VF. These risk factors were consistent when recurrent viremia was examined as the outcome; in this longitudinal analysis age at DTG start was also associated, with higher risk among children aged <6 years and adolescents aged 12–<15 years compared to those aged 6–<12 years.

Delivering effective ART to CALHIV in low- and middle-income settings remains challenging due to delayed diagnosis, formulation limitations, caregiver dependence, and adherence difficulties related to stigma, mental health, and transition of care and risk-taking behaviours factors – all factors consistently associated with VF.^23^, ^24^, ^25^ CALHIV who are ART-experienced with detectable VL at DTG start require careful evaluation for confirmed failure and consideration of resistance testing to guide optimal nucleoside reverse transcriptase inhibitor (NRTI) selection and avoid functional DTG monotherapy.^26^ Our findings are broadly consistent with previous reports from sub-Saharan Africa, where VS on DTG-based regimens among CALHIV ranged from 79 to 93% at various timepoints.^7^ We also observed the highest suppression among those who were ART-experienced and virally suppressed at DTG start, similar to findings from the European Pregnancy and Pediatric Infections Cohort Collaboration (EPPICC).^27^

Few studies have reported the cumulative incidence of viremia or confirmed VF over time among CALHIV on DTG. The EPPICC cohort of approximately 1200 CALHIV on DTG across Europe and Thailand (including 2 centres in Thailand) reported cumulative incidence of confirmed VF (defined as 2 consecutive VL > 400 copies/mL) of 4.3% (95% CI, 3.1–6.1) at week 96 and 8.3% (95% CI, 6.2–11.1) at week 144, with markedly higher incidence among those ART-experienced and viremic (VL ≥ 200 copies/mL) at DTG start. Although VF events were insufficient for a fully adjusted analyses in EPPICC, partially adjusted models similarly identified ART-experienced and viremic status at DTG start and female sex as risk factors. In our study, cumulative incidence of confirmed VF by 2 and 3 years after DTG start was higher (11% and 14%, respectively), despite use of a higher failure threshold. However, the median duration of follow-up in our cohort is limited to 1.5 years, and longer follow-up is needed to assess durability of virologic response.

The association between female sex and VF was somewhat unexpected. Similar findings have been reported in the EPPICC, an adult Thai cohort, and a real-world study among youth with HIV,^10^^,^^27^, ^28^, ^29^ whereas sex has not been associated with VF in paediatric cohorts in the African settings.^30^ These differences may reflect context-specific factors and warrants further investigation. In Thailand, DTG-based ART was widely implemented from 2021; although early teratogenicity concerns may have delayed uptake among older female adolescents, nearly halfof participants initiating DTG from 2022 onwards were female, suggesting these concerns had largely been addressed, with no observed differences in VL monitoring by sex.^19^^,^^31^

We also found that younger children (<6 years), and adolescents aged 12–<15 years had higher risk of VF or recurrent viremia compared with children aged 6–<12 years, consistent with previous studies.^9^^,^^12^^,^^13^^,^^27^^,^^30^ These findings indicate that both very young children and adolescents remain vulnerable, underscoring the need for age-tailored adherence support and closer VL monitoring.^12^ Notably, recurrent viremia was associated with subsequent confirmed VF, consistent with findings from a multicentre cohort in Tanzania showing increased VF risk among CALHIV with prior low-level viraemia (VL 50–999 copies/mL) on DTG-based ART.^32^

Low CD4 cell counts at DTG start was a strong predictor of poorer outcomes in our cohort. CALHIV with CD4 <200 cells/mm^3^ had the highest risk for viremia and confirmed VF and were nearly four times more likely to experience recurrent viremia, consistent with findings from previous studies.^27^^,^^33^ These findings reinforce that immune status at ART start remains a critical determinant of treatment outcomes, even with DTG-based regimens. This highlights the importance of CD4 monitoring to identify those immunocompromised who would benefit from enhanced clinical assessment and closer virologic monitoring. Across high-risk groups, adherence counselling remain a key component of optimising treatment outcomes among CALHIV.

This study has several strengths, including the use of national-level data from Thailand’s UHC program, capturing longitudinal ART and laboratory data for CALHIV receiving care within the public-sector during the DTG rollout period. The large sample size and comprehensive follow-up allowed robust estimation of DTG outcomes and identification of key risk factors. Inclusion of both viremia and confirmed VF allowed for more nuanced interpretation of programmatic virologic control. Limitations include reliance on routinely collected clinical records, which may be subject to incomplete information and potential misclassification of VL status at DTG start among ART-experienced individuals with infrequent VL monitoring. Lack of data on potential confounders such as adherence, resistance, psychosocial and socioeconomic factors, not available in the NAP database may have resulted in residual confounding. Information on specific opportunistic infections and concomitant treatments, including rifampicin use for tuberculosis, was unavailable. Finally, CALHIV treated exclusively in private facilities or enrolled in clinical trials were not captured, although this likely represents a small proportion of the paediatric population on DTG.

In summary, DTG-based regimens were effective for most CALHIV in Thailand, with high rates of viral suppression observed, particularly among those suppressed prior to DTG start. However, substantial risk of virologic failure persists in specific subgroups, especially ART-experienced individuals with detectable VL at DTG start. These findings highlight the importance of thorough ART history review, careful assessment of DTG transition, and targeted adherence support to optimise DTG treatment outcomes.

## Contributors

S.T., W.N.S. and I.J.C created the study concept and study design. S.T and S.C. was responsible for data collection. S.C. S.A. and W.N.S. oversaw program implementation. S.T. conducted the data management and doing analysis. I.J.C., S.A., S.C. and W.N.S. advised on the analysis. S.T. and I.J.C. drafted the manuscript. All authors critically reviewed the manuscript and approved the manuscript for submission.

## Data sharing statement

The data that support the findings of this study are available from the corresponding author upon reasonable request. However, restrictions apply to the availability of these data, which are obtained from the Thai National AIDS Program database. Due to the confidential nature of this database, data cannot be shared publicly but may be accessed with appropriate approvals.

## Declaration of generative AI and AI-assisted technologies in the writing process

During the preparation of this work, the authors used ChatGPT (OpenAI) to support text revision and formatting. After using this tool, the authors reviewed and edited the content as needed and take full responsibility for the final version of the manuscript.

## Declaration of interests

IJC has received grants via her institution from ViiV Healthcare and Gilead for projects outside of this piece of work.
