# Supplementary material for: Virologic outcomes on Dolutegravir-based therapy among children and adolescents living with HIV in Thailand: findings from a national registry database
Source: Lancet Reg Health Southeast Asia. 2026 May 20;49:100782. doi: 10.1016/j.lansea.2026.100782 (PMC13213633; doi:10.1016/j.lansea.2026.100782)
Supplement: Supplementary Figs. S1–S3 and Tables S1–S4 [file mmc1.docx]

**Supplemental appendix**

**Supplemental Figure 1 flow chart of study populations**

**Supplemental Table 1 Characteristics of CALHIV at ART initiation and at DTG-based ART initiation by inclusion criteria status**

| **Characteristics** | **Having VL tests** | **No VL tests/Incomplete ART history** | **Total** |
| --- | --- | --- | --- |
|  | **2,395** | **769** | **3,164** |
| **At ART initiation** |  |  |  |
| **Sex** | 1,229 (51) | 422 (55) | 1,651 (52) |
| male | 1,166 (49) | 347 (45) | 1,513 (48) |
| female |  |  |  |
| **Median (IQR) age at ART initiation, years** | 8 (2-16) | 15 (4-17) | 10 (3-16) |
| **Mode of HIV acquisition** |  |  |  |
| Perinatal | 1605 (67) | 368 (48) | 1973 (62) |
| Non-Perinatal | 790 (33) | 401 (52) | 1191 (38) |
| **Year at ART initiation** |  |  |  |
| 2008-2018 | 1240 (52) | 201 (26) | 1441 (46) |
| 2019-2021 | 382 (16) | 87 (11) | 469 (15) |
| 2022-2024 | 773 (32) | 481 (63) | 1254 (40) |
| **Median pre-ART CD4 at ART initiation** | 440 (213-802) | 444 (259-726) | 441 (223-775) |
| <200 | 520 (22) | 231 (30) | 751 (24) |
| 200-<350 | 361 (15) | 136 (18) | 497 (16) |
| 350-<500 | 355 (15) | 267 (35) | 622 (20) |
| ≥ 500 | 983 (41) | 135 (18) | 1118 (35) |
| unknown | 176 (7) | 0 (0) | 176 (6) |
| **First regimen** |  |  |  |
| NNRTI+NRTIs | 1286 (54) | 212 (28) | 1498 (47) |
| PIs+NRTIs | 359 (15) | 103 (13) | 462 (15) |
| DTG-based | 736 (31) | 444 (58) | 1180 (37) |
| Others (mono-NRTIs, PI+NNRTIs, INSTs) | 14 (1) | 10 (1) | 24 (1) |
| **HIV stages** |  |  |  |
| Asymptomatic HIV | 1801 (75) | 616 (80) | 2417 (76) |
| Symptomatic HIV | 258 (11) | 66 (9) | 324 (10) |
| AIDS | 336 (14) | 87 (11) | 423 (13) |
| **At DTG-based ART initiation** |  |  |  |
| **Median (IQR) age at starting DTG, years** | 15 (12-17) | 16 (13-17) | 15 (12-17) |
| < 6 | 119 (5) | 69 (9) | 188 (6) |
| 6-<12 | 421 (18) | 100 (13) | 521 (16) |
| 12-<15 | 536 (22) | 105 (14) | 641 (20) |
| ≥15 | 1319 (55) | 495 (64) | 1814 (57) |
| **Median (IQR) CD4 at starting DTG, cells/mm^3^** | 540 (324-805) | 432 (268-626) | 512 (309-773) |
| <200 | 243 (10) | 83 (12) | 335 (11) |
| 200-<350 | 253 (11) | 81 (12) | 343 (11) |
| 350-<500 | 304 (13) | 114 (17) | 431 (14) |
| ≥ 500 | 991 (41) | 173 (25) | 1186 (37) |
| unknown | 604 (25) | 231 (34) | 869 (27) |
| **Having opportunistic infection** |  |  |  |
| Yes | 189 (8) | 42 (5) | 231 (7) |
| No | 2206 (92) | 727 (95) | 2933 (93) |
| **Year at DTG start** |  |  |  |
| 2017-2018 | 26 (1) | 6 (1) | 32 (1) |
| 2019-2021 | 129 (5) | 17 (2) | 146 (5) |
| 2022-2024 | 2240 (94) | 746 (97) | 2986 (94) |
| **First DTG regimen** |  |  |  |
| DTG+NRTIs | 2350 (98) | 755 (98) | 3105 (98) |
| DTG+PIs | 45 (2) | 14 (2) | 59 (2) |
| **Median (IQR) pre-started DTG of log 10 VL, copies/mL** | 1 (1-2) | 2 (1-3) | 1 (1-2) |
| **ART and VL status at DTG-based ART initiation** |  |  |  |
| ART Naïve | 736 (31) | 444 (58) | 1180 (37) |
| ART experienced with VL < 50 copies/mL | 1118 (47) | 83 (11) | 1201 (38) |
| ART experienced with VL ≥ 50 copies/mL | 382 (16) | 65 (8) | 447 (14) |
| ART experienced with unknown VL | 159 (7) | 177 (23) | 336 (11) |
| **Duration on ART before started DTG, years** | 4 (0-9) | 0 (0-4) | 2 (0-9) |
| **Duration on DTG based ART, years** | 2 (1-2) | 0 (0-1) | 1 (1-2) |
| **Death, N(%)** | 23 (1) | 28 (4) | 51 (2) |
| **Lost to follow-up, N (%)** | 73 (3) | 104 (14) | 177 (6) |

Characteristics are summarized as n (%) or median [IQR]. Abbreviations: ART, antiretroviral therapy; NNRTI, non-nucleoside reverse transcriptase inhibitor; PI, protease inhibitor; DTG, Dolutegravir; NRTIs, nucleoside reverse transcriptase inhibitors; VL, viral load. Presented as n (%) for categorical data and median [interquartile range, IQR] for continuous data. Opportunistic infections (OIs) reflect diagnoses recorded prior to or at the time of DTG initiation

**Supplemental Table 2 Characteristics of CALHIV who had ≥ 2 VL measurements by ART and viral load status (N=1,290)**

| **Characteristics** | **Naive** | **ART experienced with VL < 50 copies/mL** | **ART experienced with VL ≥ 50 copies/mL** | **ART experienced with unknown VL** | **Total** |
| --- | --- | --- | --- | --- | --- |
|  | 307 (24) | 660 (51) | 243 (19) | 80 (6) | 1290 |
| **At ART initiation** |  |  |  |  |  |
| **Sex** |  |  |  |  |  |
| male | 176 (57) | 316 (48) | 94 (39) | 36 (45) | 622 (48) |
| female | 131 (43) | 344 (52) | 149 (61) | 44 (55) | 668 (52) |
| **Median (IQR) age at ART initiation, years** | 16 (15-17) | 5 (1-9) | 5 (2-9) | 2 (0-7) | 7.00 (2.00-15.00) |
| **Mode of HIV acquisition** |  |  |  |  |  |
| Perinatal | 67 (22) | 571 (87) | 222 (91) | 78 (98) | 938 (73) |
| Non-Perinatal | 240 (78) | 89 (13) | 21 (9) | 2 (2) | 352 (27) |
| **Year at ART initiation** |  |  |  |  |  |
| 2008-2018 | 14 (5) | 500 (76) | 185 (76) | 74 (92) | 773 (60) |
| 2019-2021 | 22 (7) | 136 (21) | 44 (18) | 6 (8) | 208 (16) |
| 2022-2024 | 271 (88) | 24 (4) | 14 (6) | 0 (0) | 309 (24) |
| **Median pre-ART CD4 at ART initiation** |  |  |  |  |  |
| <200 | 62 (20) | 146 (22) | 78 (32) | 11 (14) | 297 (23) |
| 200-<350 | 71 (23) | 82 (12) | 27 (11) | 9 (11) | 189 (15) |
| 350-<500 | 65 (21) | 77 (12) | 28 (12) | 10 (12) | 180 (14) |
| ≥ 500 | 94 (31) | 293 (44) | 87 (36) | 38 (48) | 512 (40) |
| unknown | 15 (5) | 62 (9) | 23 (9) | 12 (15) | 112 (9) |
| **First regimen** |  |  |  |  |  |
| NNRTI+NRTIs | 0 (0) | 535 (81) | 192 (79) | 53 (66) | 780 (60) |
| PIs+NRTIs | 0 (0) | 118 (18) | 50 (21) | 25 (31) | 193 (15) |
| DTG-based | 307 (100) | 0 (0) | 0 (0) | 0 (0) | 307 (24) |
| others | 0 (0) | 7 (1) | 1 (0) | 2 (2) | 10 (1) |
| **HIV stages** |  |  |  |  |  |
| Asymptomatic HIV | 206 (67) | 392 (59) | 129 (53) | 51 (64) | 778 (60) |
| Symptomatic HIV | 25 (8) | 51 (8) | 13 (5) | 4 (5) | 93 (7) |
| AIDS | 76 (25) | 217 (33) | 101 (42) | 25 (31) | 419 (32) |
| **At starting with DTG** |  |  |  |  |  |
| **Median (IQR) age at starting DTG, years** | 16.00 (15.00-17.00) | 14.00 (11.00-16.00) | 14.00 (11.00-16.00) | 13.00 (9.00-15.00) | 15.00 (12.00-16.00) |
| < 6 | 8 (3) | 19 (3) | 21 (9) | 8 (10) | 56 (4) |
| 6-<12 | 18 (6) | 164 (25) | 44 (18) | 20 (25) | 246 (19) |
| 12-<15 | 41 (13) | 195 (30) | 58 (24) | 26 (32) | 320 (25) |
| ≥15 | 240 (78) | 282 (43) | 120 (49) | 26 (32) | 668 (52) |
| **Median (IQR) CD4 at starting DTG, cells/mm3** |  |  |  |  |  |
| <200 | 62 (20) | 13 (2) | 53 (22) | 0 (0) | 128 (10) |
| 200-<350 | 69 (22) | 24 (4) | 30 (12) | 3 (4) | 126 (10) |
| 350-<500 | 61 (20) | 65 (10) | 27 (11) | 5 (6) | 158 (12) |
| ≥ 500 | 85 (28) | 385 (58) | 82 (34) | 37 (46) | 589 (46) |
| unknown | 30 (10) | 173 (26) | 51 (21) | 35 (44) | 289 (22) |
| **Having opportunistic infection at DTG-based ART initiation** |  |  |  |  |  |
| Yes | 6 (2) | 62 (9) | 28 (12) | 13 (16) | 109 (8) |
| No | 301 (98) | 598 (91) | 215 (88) | 67 (84) | 1181 (92) |
| **Year at DTG-based ART initiation** |  |  |  |  |  |
| 2017-2018 | 11 (4) | 4 (1) | 4 (2) | 4 (5) | 23 (2) |
| 2019-2021 | 22 (7) | 47 (7) | 23 (9) | 16 (20) | 108 (8) |
| 2022-2024 | 274 (89) | 609 (92) | 216 (89) | 60 (75) | 1159 (90) |
| **First DTG regimen** |  |  |  |  |  |
| DTG+NRTIs | 307 (100) | 649 (98) | 229 (94) | 74 (92) | 1259 (98) |
| DTG+PIs (+/-NRTIs) | 0 (0) | 11 (2) | 10 (6) | 6 (7) | 31 (2) |
| **Median (IQR) pre-started DTG of log 10 VL, copies/mL** |  |  |  |  |  |
| ≤50 | 206 (67) | 660 (100) | 0 (0) | 0 (0) | 866 (67) |
| >50 | 82 (27) | 0 (0) | 243 (100) | 0 (0) | 325 (25) |
| unknown | 19 (6) | 0 (0) | 0 (0) | 80 (100) | 99 (8) |
| <1000 | 226 (81) | 660 (100) | 27 (14) | 0 (0) | 913 (76) |
| ≥1000 | 34 (12) | 0 (0) | 160 (86) | 0 (0) | 194 (16) |
| unknown | 19 (7) | 0 (0) | 0 (0) | 80 (100) | 99 (8) |
| **Duration on ART before started DTG,** | 0.00 (0.00-0.00) | 7.71 (3.68-10.89) | 7.41 (3.55-10.52) | 8.69 (5.53-11.41) | 5.56 (0.27-9.94) |
| **Duration on DTG based ART, years** | 1.74 (1.30-2.05) | 1.85 (1.64-2.05) | 1.80 (1.40-2.10) | 2.01 (1.72-2.36) | 1.84 (1.54-2.07) |
| **Death, N(%)** | 2 (1) | 1 (0) | 5 (2) | 1 (1) | 9 (1) |
| **Mortality rate per 100 person-year** | 0.2 (0.05-0.8) | 0.03 (0-0.22) | 0.81 (0.34-1.95) | 0.5 (0.07-3.53) | 0.18 (0.09-0.35) |
| **Lost to follow-up, N (%)** | 2 (1) | 1 (0) | 3 (1) | 1 (1) | 7 (1) |

Characteristics are summarized as n (%) or median [IQR]. Abbreviations: ART, antiretroviral therapy; NNRTI, non-nucleoside reverse transcriptase inhibitor; PI, protease inhibitor; DTG, Dolutegravir; NRTIs, nucleoside reverse transcriptase inhibitors; VL, viral load. Presented as n (%) for categorical data and median [interquartile range, IQR] for continuous data. Opportunistic infections (OIs) reflect diagnoses recorded prior to or at the time of DTG initiation.

**Supplemental Table 3 Probability of viremia and confirmed VF by prior ART experience with VL status at year 1, 2, and 3**

| **ART experienced with VL status** | **Year at risk** | | |
| --- | --- | --- | --- |
|  | **1** | **2** | **3** |
| **Probability of viremia (N=2,395)** |  |  |  |
| Overall | 0.14 (0.13-0.16) | 0.29 (0.25-0.33) | 0.37 (0.31-0.44) |
| Naive | 0.14 (0.11-0.17) | 0.29 (0.23-0.37) | 0.37 (0.28-0.47) |
| ART experienced with VL < 50 copies/mL | 0.02 (0.01-0.03) | 0.17 (0.11-0.24) | 0.39 (0.22-0.63) |
| ART experienced with VL ≥ 50 copies/mL | 0.57 (0.52-0.63) | 0.75 (0.66-0.83) | 0.75 (0.66-0.83) |
| ART experienced with unknown VL | 0 (0-0) | 0.09 (0.04-0.20) | 0.14 (0.06-0.30) |
|  |  |  |  |
| **Probability of confirmed VF (N=1,290)** |  |  |  |
| Overall | 0.04 (0.03-0.05) | 0.11 (0.09-0.14) | 0.14 (0.10-0.20) |
| Naive | 0.02 (0.01-0.05) | 0.04 (0.02-0.08) | 0.12 (0.05-0.28) |
| ART experienced with VL < 50 copies/mL | 0 (0-0) | 0.04 (0.02-0.09) | 0.04 (0.02-0.09) |
| ART experienced with VL ≥ 50 copies/mL | 0.17 (0.13-0.22) | 0.42 (0.33-0.52) | 0.47 (0.35-0.61) |
| ART experienced with unknown VL | 0 (0-0) | 0.04 (0.01-0.25) | 0.04 (0.01-0.25) |

**Supplemental Figure 2 probability of viremia by age at DTG-based ART initiation**

**
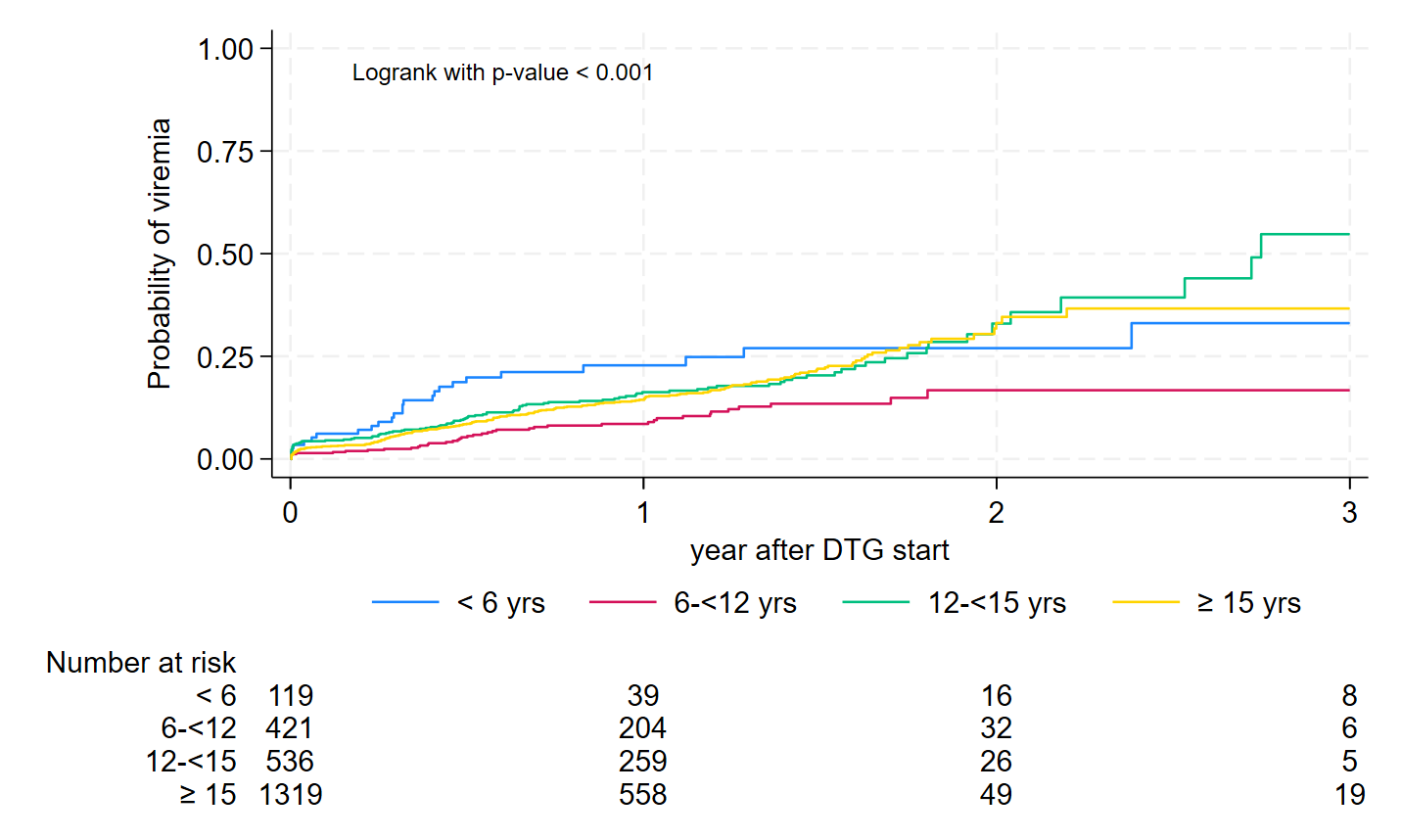
**

**Supplemental Figure 3 probability of confirmed VF; a) overall and by sex at birth and b) by prior ART experience with VL status and by CD4 at DTG-based ART initiation**

**
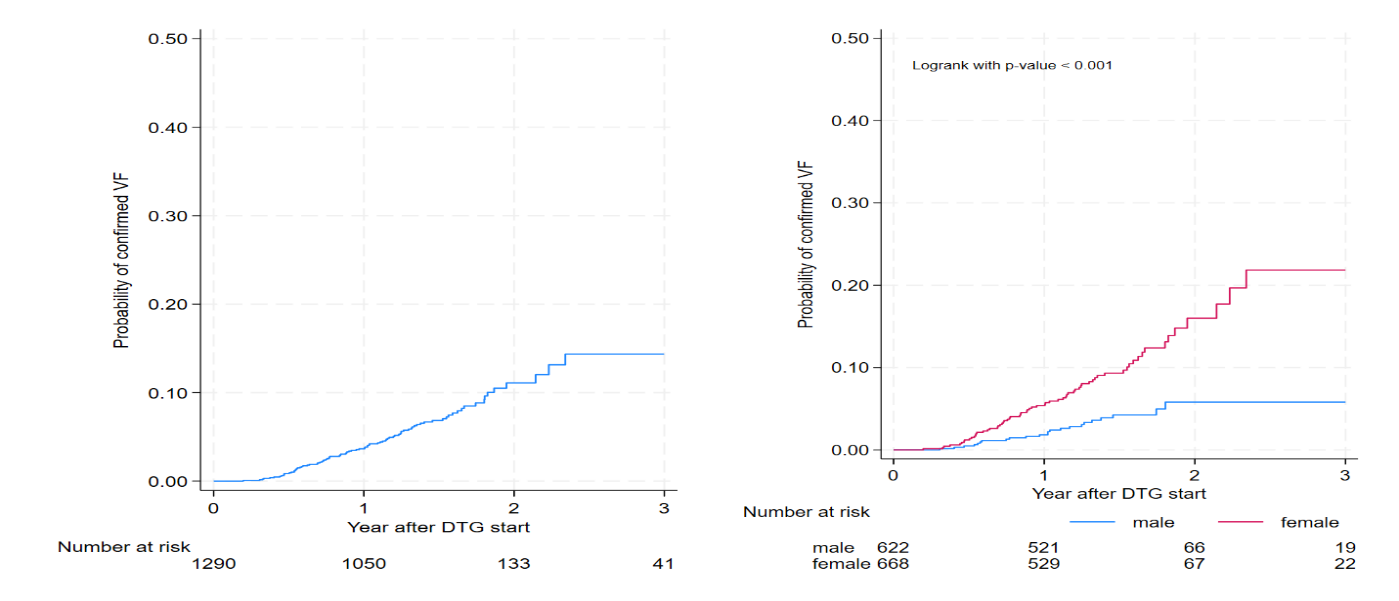
**

**a) overall and by sex at birth**


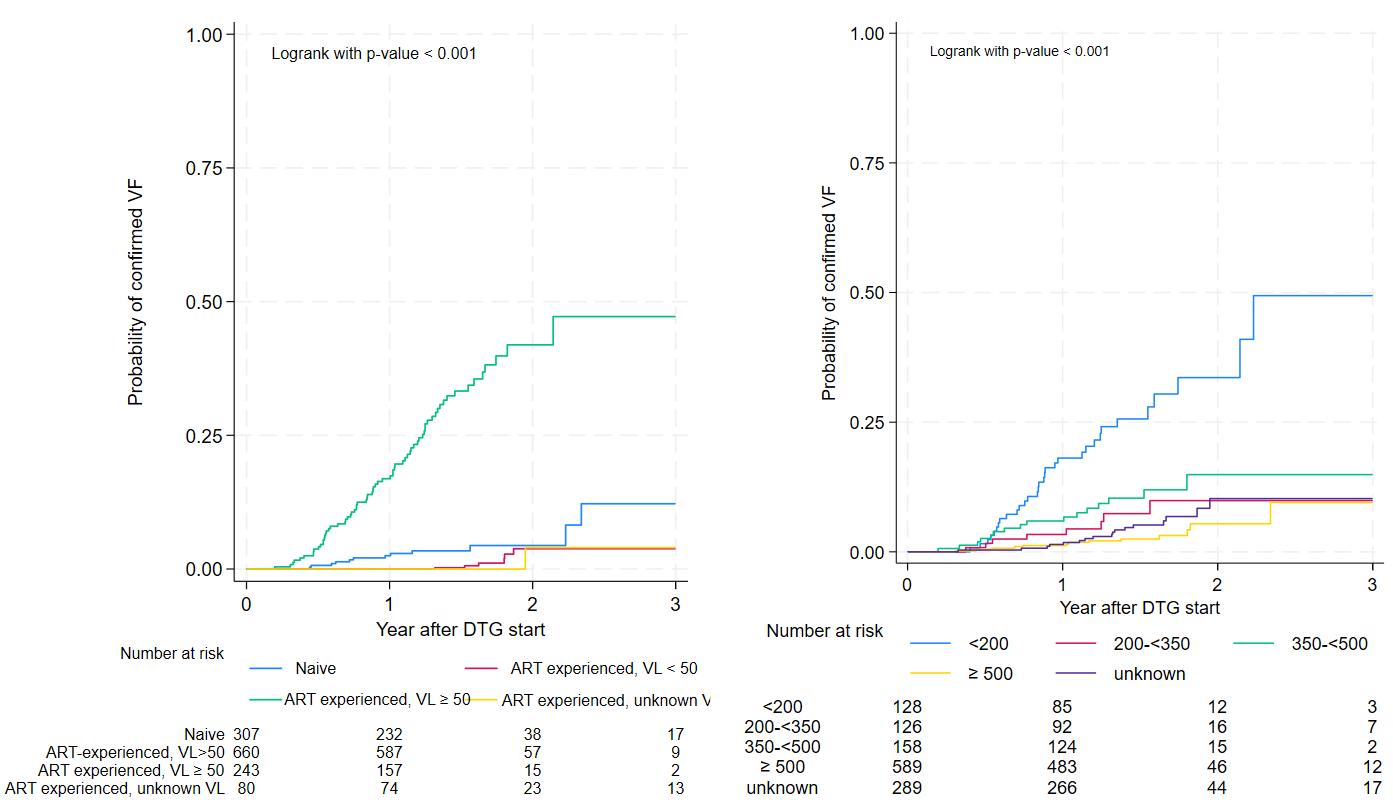


**b) by prior ART experience with VL status and by CD4 at DTG-based ART initiation**

# **Supplementary Table 4. RECORD Checklist for Observational Studies Using Routinely Collected Health Data**

| Item No. | RECORD Item | Description | Location in Manuscript |
| --- | --- | --- | --- |
| 1.1 | Type of data | Type of routinely collected data and database name specified | Title; Abstract; Methods (Study design, Data management) |
| 1.2 | Geographic region and timeframe | Study conducted in Thailand using National AIDS Program (NAP) database; period up to August 2024 | Abstract; Methods (Study design) |
| 1.3 | Data linkage | Linkage with National Death Registry stated | Methods (Data management) |
| 6.1 | Study population selection | Inclusion: age <18 at DTG-based ART initiation and ≥1 VL after DTG; Exclusion: incomplete ART history or no post-DTG VL | Methods (Study design); Supplemental Figure 1 |
| 6.2 | Validation of codes/algorithms | Clinical definitions based on Thai National Guidelines and WHO definitions; no additional validation studies performed | Methods (Definitions and outcomes) |
| 6.3 | Data linkage process | NAP linked to National Death Registry; flow diagram provided | Methods; Supplemental Figure 1 |
| 7.1 | Definition of variables | Outcomes (VS, viremia, confirmed VF), exposures, covariates, CD4 strata clearly defined | Methods (Definitions and outcomes; Statistical analysis) |
| 12.1 | Data access | Authors had access to de-identified individual-level data from NAP | Methods (Data management) |
| 12.2 | Data cleaning | Internal consistency checks, duplication checks, chronological validation performed | Methods (Data management) |
| 12.3 | Linkage methods | Routine person-level linkage to National Death Registry managed by NHSO | Methods (Data management) |
| 13.1 | Participant selection reporting | Detailed selection and exclusions described; flow diagram provided | Results (Study population); Supplemental Figure 1 |
| 19.1 | Limitations of routinely collected data | Discussion of missing VL data, reporting delays, absence of adherence/resistance data, residual confounding | Discussion (Limitations section) |
| 22.1 | Accessibility of data | Data available upon reasonable request; restrictions apply due to confidentiality | Data Availability Statement |
| 22 | Funding | Funding source and role of funder stated | Funding section |
